# Tunable Emission Wavelength Stacked InAs/GaAs Quantum Dots by Chemical Beam Epitaxy for Optical Coherence Tomography

**DOI:** 10.3390/ma9070511

**Published:** 2016-06-24

**Authors:** Bouraoui Ilahi, Jihene Zribi, Maxime Guillotte, Richard Arès, Vincent Aimez, Denis Morris

**Affiliations:** 1 Université de Sherbrooke, Laboratoire Nanotechnologies Nanosystèmes (LN2)–CNRS UMI-3463, Institut Interdisciplinaire d’Innovation Technologique (3IT), Sherbrooke, QC J1K OA5, Canada; Jihene.Zribi@USherbrooke.ca (J.Z.); Maxime.Guillotte@USherbrooke.ca (M.G.); Richard.Ares@USherbrooke.ca (R.A.); Vincent.Aimez@USherbrooke.ca (V.A.); Denis.Morris@USherbrooke.ca (D.M.); 2 King Saud University, Department of Physics & Astronomy, College of Sciences, Riyadh 11451, Saudi Arabia

**Keywords:** InAs quantum dots, In-flush, chemical beam epitaxy, SLD, OCT

## Abstract

We report on Chemical Beam Epitaxy (CBE) growth of wavelength tunable InAs/GaAs quantum dots (QD) based superluminescent diode’s active layer suitable for Optical Coherence Tomography (OCT). The In-flush technique has been employed to fabricate QD with controllable heights, from 5 nm down to 2 nm, allowing a tunable emission band over 160 nm. The emission wavelength blueshift has been ensured by reducing both dots’ height and composition. A structure containing four vertically stacked height-engineered QDs have been fabricated, showing a room temperature broad emission band centered at 1.1 µm. The buried QD layers remain insensitive to the In-flush process of the subsequent layers, testifying the reliability of the process for broadband light sources required for high axial resolution OCT imaging.

## 1. Introduction

The near IR optical imaging system has been demonstrated as a penetrative and non-invasive tool for medical applications [1,2]. Particularly, Optical Coherence Tomography (OCT) has received an increasing interest owing to its ability to form 3D images down to 2 mm in depth beneath the biological tissue’s surface [3], prompting a great deal of potential as a high-resolution real-time microscopic optical-imaging technique, suitable for imaging breast cancer tumor margins intraoperatively [2]. While, ultra high resolution in the submicrometer scale has been demonstrated with OCT using a Femtosecond laser [4], this optical source is costly and relatively complex to operate, imposing a major challenge to widespread clinical applications [5]. Superluminescent diode (SLD) appears to be as an alternative solution. Indeed, compared to solid state femtosecond laser, this optical source is much cheaper and easier to maneuver. Furthermore, the SLD based on a self-assembled InAs/GaAs quantum dots (QDs) system turns out to be an appropriate light emitting source for OCT imaging [6,7,8]. Indeed, it is a tunable wavelength source that emits in a range corresponding to the minimum scattering losses into the skin tissue [9]. Additionally, the large natural size distribution within the QD ensemble [10,11] is an asset for the realization of a broadband emission source that is required to improve the axial resolution of the imaging system [12]. Even larger emission and more controllable wavelength changes can be induced using QD engineering approaches, based on thermal annealing, and ion implantation techniques [13,14,15]. The indium-flush process is one of the most powerful approaches for in situ control of the QD structural properties. This process has been successively used to tune the SLD bandwidth [6,7,8] grown by conventional epitaxial growth techniques such as molecular beam epitaxy (MBE) and metalorganic chemical vapour deposition (MOCVD). The suitability of Chemical Beam Epitaxy (CBE) in the fabrication of wavelength tunable InAs QD for SLD has not been reported yet.

This paper, reports on the investigation of the effects of indium-flush process to engineer the InAs/GaAs QD size using CBE, and the feasibility of a SLD active layer with a controllable broad emission band for OCT.

## 2. Results

### 2.1. Samples Growth

The investigated InAs QD layers (1.9 ML nominal thickness) were deposited by CBE at 465 °C with a growth rate of 0.159 ML/s on 300 nm thick GaAs buffer layer. After a growth interruption for 60 s under AsH3 flux, the QDs were capped in two-step process. A first cap layer with variable thickness (h), ranging from 5 to 2 nm, was deposited at 465 °C. The sample is then annealed under an AsH3 flux at 600 °C for 10 min in order to evaporate the uncovered QD tip. The process is completed by growing the remaining 30 nm thick GaAs cap layer.

A series of single layer QD samples were fabricated with different GaAs cap layer thicknesses (from 2 to 5 nm). A structure containing four vertically stacked height-engineered QDs, separated by a 30-nm thick GaAs spacer layer, has also been fabricated in the same conditions. The QD heights in this sample are expected to be 5, 4, 3 and 2.5 nm.

### 2.2. Height Truncation of Single QD Layer

**Figure 1:**
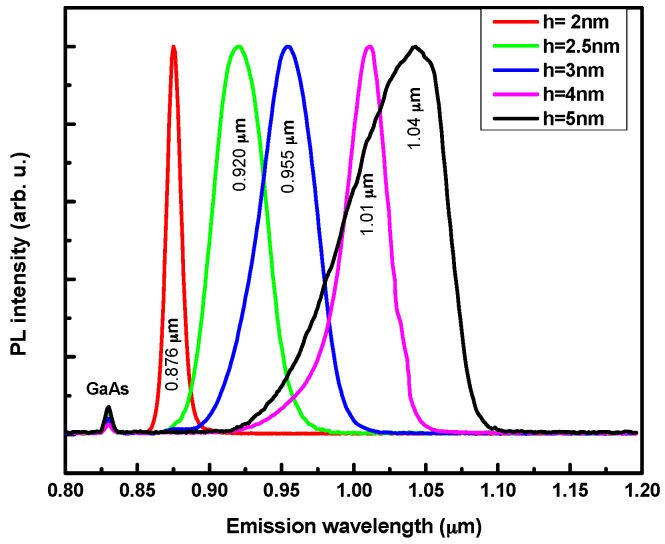
Normalized photoluminescence spectra measured at 19 K with 0.48 mW excitation power for samples with truncated quantum dot (QD) heights ranging from 2 nm up to 5 nm.

Figure 1 shows the low-temperature photoluminescence (PL) spectra taken at low excitation power (0.48 mW) from samples with GaAs capping layer thickness ranging from 5 nm to 2 nm. The conventional QD emission wavelength is around 1.04 µm at 19 K. The In-flush process produced no significant changes in the PL spectra for samples having a first cap layer thicker than 5 nm. However, for cap layer thicknesses below 5 nm, the QD emission bands get blue shifted ensuring a tunable emission wavelength over 160 nm. Indeed, during the in situ annealing step, aiming to evaporate the uncovered QD tip, the In composition also gets reduced due to excess In evaporation. The amount of evaporated indium is proportional to the exposed area, which increases with decreasing QD height [15]. A homogenization of the dot size dispersion, perceptible through the PL linewidth shrinkage has been found to occur for engineered QD with a height below 5 nm. Furthermore, the QD optical properties are strongly affected by reducing the GaAs cap layer down to 2.5 nm, showing a drastic change in their shape and average aspect ratio. The observed evolution can be interpreted in terms of intermixing induced changes of the QD composition.

No excited states emission have been observed for h = 2 nm. Instead of that, a blue shift of 12 meV has been found to occur simultaneously with a PL peak linewidth enhancement, when the excitation power increases. For this specific case, the QD states are expected to be quasi resonant with the wetting layer continuum states.

The investigation of height truncated QD in separated single layers shows that the In-flush technique can be reliably employed in CBE to tune the QD emission wavelength. However, the fabrication of QD-based SLD requires vertical stacking of engineered QD with different heights to enhance the emission bandwidth of this source. In this context, we have fabricated a structure containing 4 QD layers with different heights in the same growth run using the experimental conditions described above. The QD heights are 5, 4, 3 and 2.5 nm.

### 2.3. Staked Layers of QD with Different Heights

**Figure 2:**
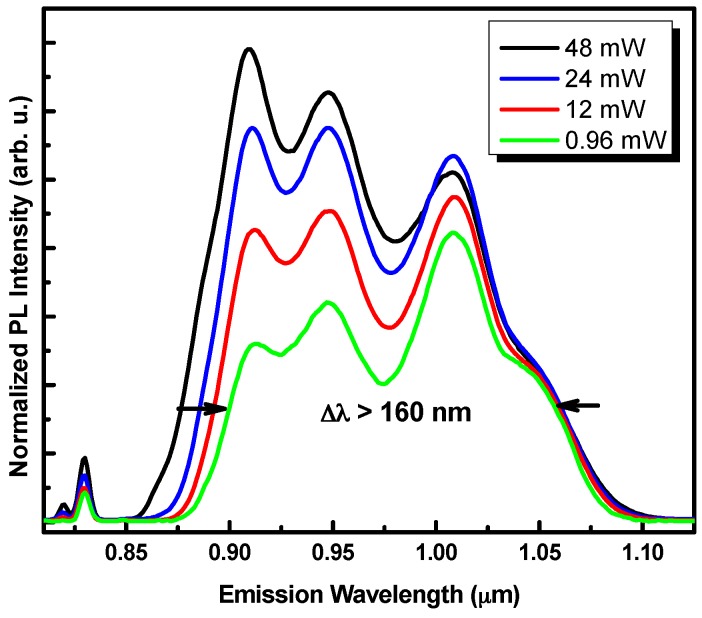
Low-temperature (19 K) photoluminescence spectra as a function of the excitation power for vertically stacked height engineered QD. The spectra are normalized to the 5 nm height QD PL peak’s intensity.

Figure 2 shows the low-temperature PL spectra at various excitation powers obtained for the 4 QD layers sample. At the lowest excitation power, the spectrum shows four distinct emission bands corresponding to the QD ground state emission bands of each QD plane of the structure. When the excitation power increases, the relative intensity of the emission bands at lower wavelengths increases significantly. This behavior is explained by the contribution of extra optical transitions between the excited states of the bigger quantum dots.

**Figure 3:**
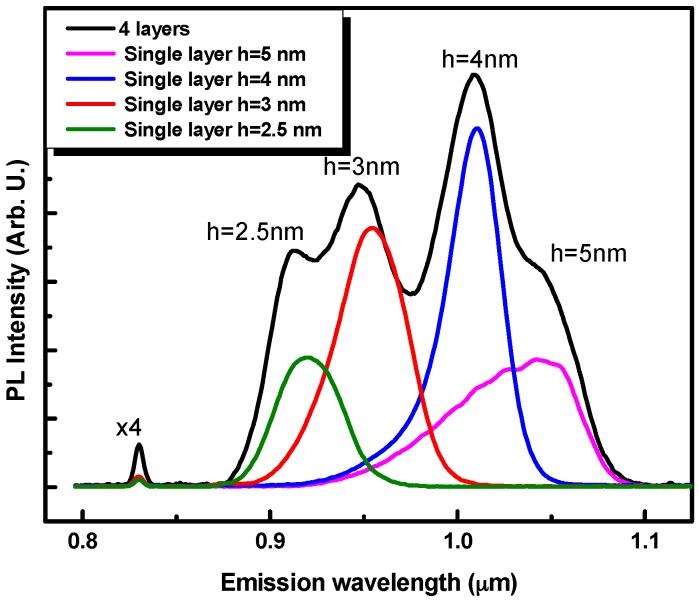
Photoluminescence spectra measured at 19 K with 0.48 mW excitation power for samples with single layers of truncated QD height ranging from 2.5 nm up to 5 nm, in addition to the multiple peaks PL spectrum from the stacked QD layers.

To check whether the In-flush processing of the upper QD layer affects the buried ones, low excitation power PL spectra of single QD layers with different heights are plotted in the Figure 3 together with the multipeak spectrum from vertically stacked layers. Each emission band of the four QD layer spectrums is found to match the corresponding emission band (for a given QD height) of the single QD layer spectra. This good matching indicates that the successive in situ annealing step do not alter the QD properties of the buried QD layers. These results testify that the chosen growth conditions insure a good stability of the QD structure.

**Figure 4:**
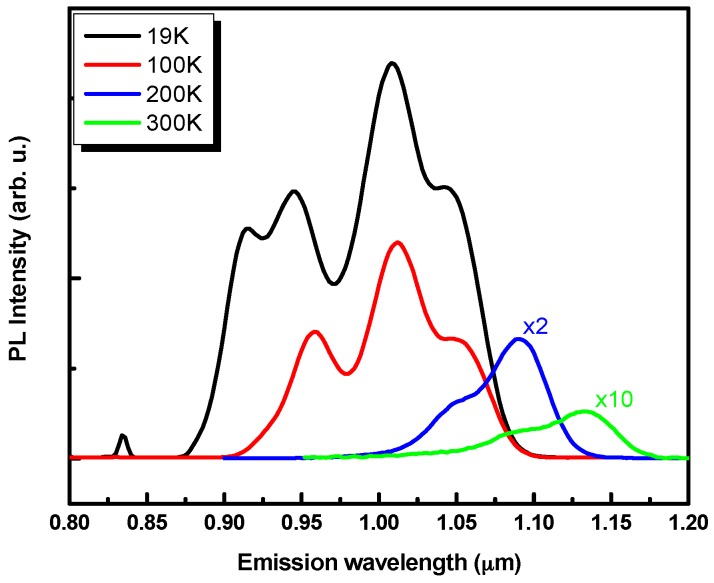
Temperature-dependent photoluminescence spectra from vertically stacked QD with truncated height ranging from 2.5 nm to 5 nm. The spectra are measured with 0.96 mW excitation power.

In order to get more information allowing an accurate assessment of the expected SLD performance, the multiple QD layers’ PL is evaluated as a function of temperature. Figure 4 shows the PL spectra at different temperature. In addition to the obvious redshift induced by the band gap shrinkage, the spectra linewidth is found to be reduced when the temperature increases. The observed linewidth shrinkage is mainly due to the quenching of the high energy emission bands (PL coming from the smaller QD) caused by carrier thermal activation mechanisms. Indeed, the QD with the smallest height also have shallow carriers’ confining potential. At 300 K, photocarriers that are easily extracted from the smaller QD, will populate the states of bigger QD. This phenomenon could be minimized by a better fabrication design, consisting in using cladding layers with higher bandgap materials.

The central wavelength (λ_0_) and bandwidth of SLD (Δλ) are critical for improving the performance of the OCT systems [13]. λ_0_ should be within the NIR optical window, which corresponds to the range of wavelengths where light scattering and absorption by water and oxyhemoglobin (main constituents of the skin) are minimal. The axial resolution of the OCT image is evaluated using this expression $0.44\lambda_{0}^{2}/\Delta \lambda$ [13]. At 300 K, the central emission wavelength of our multi-layer QD sample is around 1.1 µm, which is within the NIR optical window of interest for the imaging of biological samples [16]. By expecting that the emission bandwidth (Δλ) of 160 nm, observed at 19 K, can be preserved at higher temperature, the expected axial resolution of OCT images obtained using a height engineered QD structure inserted in the active layer of a SLD light source, will be 3.3 µm. Such characteristics can be useful for OCT preoperative imaging of tumor thickness (Basal Cell Carcinoma) [17].

Using an extra QD plane with height as small as 2 nm it is possible to further increase the emission bandwidth, by almost 50 nm (see Figure 1) towards the low wavelength side of the spectrum. In order to extend the emission band towards higher wavelengths, which is desired to further reduce light scattering and to improve the axial resolution, it will be necessary to use strain reducing layers, such as InGaAs [18] and InGaAsN [19].

## 3. Materials and Methods

The samples’ growth was performed in a vacuum Generator V90F CBE chamber using TMIn, TEGa and cracked arsine (AsH3) as precursors.

The PL measurements have been carried out, between 19 and 300 K, using a 1-m spectrometer (SPEX model) with a liquid nitrogen-cooled Ge detector. A 532-nm diode laser with a 48-mW power has been used as an excitation source. The excitation beam was focused on a spot diameter (1/e) of about 100 µm. PL signals were recorded using conventional lock-in detection.

## 4. Conclusions

Height engineered InAs/GaAs QD have been fabricated by CBE using the In-flush process ensuring a controllable bandwidth higher than 160 nm. The employed in situ QD height truncation process is found to be practical for the growth of vertically stacked height engineered QD. We demonstrated superluminescent diode’s active layer having a linewidth broadening higher than 160 nm and central wavelength around 1.1 µm. The theoretically expected axial resolution is less than 3.3 µm, which is suitable for OCT imaging.

## Abbreviations

The following abbreviations are used in this manuscript:

CBE	Chemical Beam Epitaxy
QD	Quantum dots
SLD	Superluminescent DiodeDirectory of open access journals
OCT	Optical Coherence Tomography
PL	Photoluminecsence
TMIn	Trimethylindium
TEGa	Triethylgallium
